# PEComas: current concepts in diagnosis, molecular pathways, and emerging treatments

**DOI:** 10.1093/oncolo/oyag134

**Published:** 2026-04-08

**Authors:** Giada Del Baldo, Giuseppe Maria Milano, Mariantonietta Di Salvatore, Angela Mastronuzzi

**Affiliations:** Pediatric Hematology/Oncology and Cell and Gene Therapy, IRCCS Bambino Gesù Children’s Hospital, Rome, 00165, Italy; Pediatric Hematology/Oncology and Cell and Gene Therapy, IRCCS Bambino Gesù Children’s Hospital, Rome, 00165, Italy; Medical Oncology, Università Cattolica del Sacro Cuore, IRCCS, Rome, Italy; Pediatric Hematology/Oncology and Cell and Gene Therapy, IRCCS Bambino Gesù Children’s Hospital, Rome, 00165, Italy; Department of Life Sciences and Public Health, Università Cattolica del Sacro Cuore, Rome, 00168, Italy

**Keywords:** PEComas, rare mesenchymal tumors, risk stratification, genomic profiling, mTOR inhibitors, precision oncology

## Abstract

**Background:**

Perivascular epithelioid cell tumors (PEComas) are rare mesenchymal neoplasms with distinct histopathological, immunophenotypic, and molecular features. Described in the early 1990s, they include angiomyolipoma, lymphangioleiomyomatosis, and clear cell “sugar” tumors, and can arise in various sites, most often the kidney, uterus, lung, and soft tissues. While usually indolent, some exhibit aggressive behavior with invasion, recurrence, and metastasis.

**Methods:**

A comprehensive literature review was conducted to summarize the current understanding of PEComa biology, diagnostic approaches, risk stratification systems, and therapeutic strategies. Emphasis was placed on studies defining the molecular landscape, treatment outcomes with mTOR inhibitors, and emerging prognostic tools.

**Results:**

Molecularly, most PEComas harbor loss-of-function mutations in the TSC1 or TSC2 genes, resulting in constitutive mTOR signaling pathway activation, a central driver of tumorigenesis and a key therapeutic target. A distinct subset is defined by TFE3 gene rearrangements, exhibiting unique clinicopathologic features and suggesting an alternative, mTOR-independent oncogenic mechanism. Accurate diagnosis requires integration of morphology, immunohistochemistry, and molecular testing, while risk stratification is guided by established histologic criteria and emerging prognostic models. Surgical resection remains the standard of care for localized disease, whereas mTOR inhibitors constitute the cornerstone of systemic therapy for advanced or unresectable PEComas. Additional approaches, including immune checkpoint inhibitors, locoregional therapies, and chemotherapy, may be considered in selected cases.

**Conclusions:**

This review summarizes PEComa biology, diagnosis, prognosis, and treatment, emphasizing the need for international collaboration to improve risk stratification and personalized therapies.

Implications for Practice PEComas are ultra-rare mesenchymal tumors with highly variable clinical behavior, requiring accurate diagnosis, molecular characterization, and risk stratification. Recognition of *TSC1/TSC2* alterations is essential, as mTOR inhibitors represent the standard systemic therapy for advanced disease, with nab-sirolimus showing particularly high efficacy. Identification of *TFE3*-rearranged PEComas is critical, given their distinct biology and potential resistance to mTOR inhibition. Emerging prognostic models, such as PEC-PRO, may help guide surveillance and treatment intensity. Multidisciplinary management and referral to expert centers and registries are strongly recommended to optimize patient outcomes.

## Introduction

Perivascular epithelioid cell tumors (PEComas) represent a rare and distinctive family of mesenchymal neoplasms, first described in the early 1990s, comprise a heterogeneous group of tumors, represented by angiomyolipoma (AML), lymphangioleiomyomatosis (LAM), and clear cell “sugar” tumors of the lung.^1^ Although rare (0.12-0.24 cases per million per year), PEComas are of increasing interest due to their distinctive histology—perivascular epithelioid cells (PECs) showing melanocytic and smooth muscle features—as well as their varied anatomical distribution and complex molecular profile. The median age of patients described is 46 years (range 15-97), with a clear predominance of females.^1^

PEComas can arise in virtually any anatomical site, with a predilection for the kidney, uterus, lungs, and soft tissues. Clinically, these tumors often present as slow-growing, asymptomatic masses, but a subset exhibits aggressive behavior with local invasion, recurrence, and distant metastases.^1–3^

From a molecular standpoint, most PEComas are characterized by inactivation of the TSC1 or TSC2 genes, resulting in constitutive activation of the mTOR signaling pathway, which plays a central role in tumor pathogenesis.^4^^,^^5^

Diagnosis relies on a combination of morphological, immunophenotypic, and molecular features, according to established criteria proposed by Folpe et al.^1^ and refined in the WHO 2020 Classification of Soft Tissue Tumours.^6^ Importantly, benign PEComas do not undergo malignant transformation; rather, malignancy is determined by intrinsic histopathological features present at diagnosis according to established criteria (Folpe et al., WHO 2020).^1^^,^^6^ This distinction is critical for appropriate risk stratification and treatment.

The management of PEComas remains challenging. Surgical resection is the mainstay for localized disease, while systemic therapy, primarily with mTOR inhibitors, is used in unresectable and metastatic disease.^7^

This review aims to provide a comprehensive overview of the current understanding of PEComas, highlighting current treatment options as well as emerging research directions. Moreover, particular attention will be given to differences between sporadic and TSC-associated PEComas and to the ongoing efforts to improve risk stratification and personalize therapy in this ultra-rare tumor family.

## Histopathology

PEComas are defined histologically by the presence of PECs showing dual melanocytic and smooth muscle differentiation. Microscopically, they typically consist of epithelioid and spindle cells arranged around blood vessels, with clear to eosinophilic cytoplasm and round to oval nuclei. Malignant cases may display cytologic atypia, pleomorphism, necrosis, and vascular invasion.^8^

Immunohistochemically, PEComas consistently express melanocytic markers (HMB-45, Melan-A) and smooth muscle markers (SMA, desmin), with diffuse vimentin positivity. Other markers (CD56, CD99, S-100) may be variably expressed, while epithelial markers (cytokeratins, AE1/AE3, NKX3.1), CD117, CD34, and CD44 are typically negative. PEComas are also negative for SOX10, a sensitive and specific marker for malignant melanoma, which assists in differentiating them from morphologically similar tumors. TFE3 immunoreactivity identifies a molecularly distinct subset harboring TFE3 gene rearrangements, often characterized by an alveolar growth pattern, epithelioid cytomorphology, and absence of smooth muscle antigen expression (Table 1).^8–10^

**Table 1.** Immunohistochemical markers in PEComa diagnosis.

*Marker*	Expression in PEComa	Diagnostic utility
***HMB-45***	Positive	Melanocytic marker; highly sensitive
***Melan-A***	Positive	Melanocytic marker
***MITF***	Positive	Melanocytic transcription factor
***Smooth muscle actin (SMA)***	Positive	Smooth muscle marker
***Desmin***	Positive (variable)	Smooth muscle marker
***TFE3***	Positive in subset	Indicates TFE3 gene rearrangement
***Cytokeratins***	Negative	Helps exclude carcinoma
**S100**	Usually negative	Helps exclude melanoma

The diagnosis of PEComa requires careful exclusion of other neoplasms with overlapping morphologic or immunophenotypic features. Differential diagnoses include malignant melanoma, clear cell sarcoma, alveolar soft part sarcoma, gastrointestinal stromal tumors, rhabdomyosarcoma, myoepithelial tumors, adrenal cortical carcinoma, and certain carcinomas such as renal cell carcinoma and prostate carcinoma (Table 2). Molecular testing (NGS, FISH, RT-PCR) is essential in diagnostically challenging cases or when a TFE3-rearranged PEComa is suspected.^3^^,^^6^^,^^10–12^

**Table 2.** Immunohistochemical profile for differential diagnosis of PEComa and related tumors.

Tumor type	HMB-45	Melan-A	SMA	Desmin	TFE3	SOX10	Cytokeratin	CD117	S100
**PEComa**	+	+	+	+	+/−	−	−	−	−
**Malignant Melanoma**	+	+	−	−	−	+	−	−	+
**Clear Cell Sarcoma**	+	+	−	−	−	+	−	−	+
**GIST**	+/−	−	+/−	−	−	−	−	+	−
**Alveolar Soft Part Sarcoma**	−	−	−	−	+	−	−	−	−

+ = positive; − = negative; +/− = variable. Modified from WHO 2020 and Folpe et al., 2005.

## Molecular biology

PEComas are characterized by a heterogeneous molecular landscape that reflects distinct oncogenic pathways driving tumor development. The majority of PEComas harbor loss-of-function mutations in the TSC1 and TSC2 genes, which encode hamartin and tuberin, respectively. These proteins form a complex that negatively regulates the mammalian target of rapamycin (mTOR) signaling pathway, a critical regulator of cell growth, proliferation, and metabolism. Mutations in TSC1 or TSC2 lead to constitutive activation of mTOR signaling, promoting tumorigenesis through increased cellular proliferation and survival.^2–5^^,^^13^^,^^14^

Comprehensive genomic profiling studies have revealed that approximately 80% of PEComas without TFE3 gene rearrangements harbor TSC2 mutations, with a smaller subset carrying TSC1 mutations.^3^^,^^4^ Notably, co-occurring mutations in tumor suppressor genes such as TP53 have been identified in a significant proportion of TSC2-mutated PEComas, suggesting additional layers of genomic complexity that may influence tumor behavior and prognosis.^15^^,^^16^ Other recurrent genetic alterations include mutations in ATRX, RB1, and CDKN2A, which may contribute to genomic instability and malignant progression.^17^

A distinct molecular subset of PEComas is defined by TFE3 gene rearrangements, which occur in approximately 8%-23% of cases depending on the cohort studied.^18^ These rearrangements result in fusion transcripts involving TFE3 and various partner genes, such as SFPQ/PSF, DVL2, RRAGB, and OPHN1, among others. TFE3-rearranged PEComas exhibit unique clinicopathologic features, including a nested or alveolar growth pattern and a lack of coexisting TSC2 mutations, indicating an alternative, mTOR-independent oncogenic pathway.^19^ Immunohistochemically, these tumors show strong nuclear expression of TFE3 protein, which serves as a useful diagnostic marker.

From a biochemical standpoint, the interplay between TSC1/2, mTOR signaling, TFE3, and FLCN further refines the molecular heterogeneity of PEComas. The TSC1–TSC2 complex acts as a GAP toward RHEB, thereby inhibiting mTOR complex 1 (mTORC1). Loss of TSC1 or TSC2 leads to constitutive mTORC1 activation, promoting anabolic growth and explaining the sensitivity of these tumors to rapalogs. In contrast, TFE3-rearranged PEComas are driven by oncogenic fusions that activate MiT/TFE-dependent transcriptional programs independently of canonical TSC1/2–mTORC1 regulation. Under physiological conditions, TFE3 is regulated by mTORC1-dependent phosphorylation and cytoplasmic sequestration; however, gene rearrangements result in its constitutive nuclear localization and transcriptional activation of lysosomal and proliferative pathways. FLCN (folliculin), although rarely altered, further links these pathways. Through its role as a GAP for RagC/D GTPases, FLCN regulates amino acid–dependent mTORC1 activation and MiT/TFE transcription factor localization. Its loss may promote TFE3 nuclear translocation and synergize with TSC2 deficiency via convergent dysregulation of mTORC1 and lysosomal signaling. Collectively, these interactions support a dual oncogenic model in PEComas, with TSC1/2 loss driving mTORC1-dependent growth and TFE3 rearrangements (and rare FLCN alterations) engaging partially mTOR-independent transcriptional programs.^20–23^

Further molecular characterization has identified rare gene fusions such as HTR4-ST3GAL1 and RASSF1-PDZRN3 in isolated cases,^18^ highlighting the genetic heterogeneity of PEComas. Additionally, mutations in genes involved in DNA repair pathways, including MSH3 and ERCC2, have been observed exclusively in TSC1/2-mutated tumors, suggesting potential vulnerabilities that could be therapeutically exploited.^7^^,^^24^

The molecular dichotomy between TSC1/2-mutated and TFE3-rearranged PEComas has important therapeutic implications. While TSC1/2 mutations sensitize tumors to mTOR inhibitors such as sirolimus, everolimus, and nab-sirolimus, TFE3-rearranged tumors may require alternative targeted approaches due to their distinct biology.^10^^,^^25^^,^^26^ Emerging data also indicate that the tumor microenvironment of PEComas is relatively immune-cold compared to other malignancies like melanoma, with lower infiltration of cytotoxic T cells and B cells, which may impact the efficacy of immune checkpoint inhibitors.^27^^,^^28^

Molecular alterations in PEComa are described in Table 3.

**Table 3.** Molecular alterations in PEComa.

*Alteration*	Frequency (%)	Pathogenic role	Clinical implications
***TSC2 mutations***	∼70-80%	Loss of mTOR pathway regulation	Sensitivity to mTOR inhibitors
***TSC1 mutations***	∼10-15%	Loss of mTOR pathway regulation	Sensitivity to mTOR inhibitors
***TFE3 gene rearrangements***	8-23%	Alternative oncogenic pathway	mTOR-independent; may require different therapy
***TP53 mutations***	Variable (∼30%)	Tumor suppressor loss	Associated with aggressive behavior
***ATRX, RB1, CDKN2A mutations***	Rare to moderate	Genomic instability, cell cycle dysregulation	Potential markers of malignancy
***DNA repair gene mutations (eg, MSH3, ERCC2)***	Rare	May influence response to therapy	Potential targets for future therapies

## Cancer predisposition syndromes

The genetic landscape of PEComas includes both sporadic and syndromic forms, with a well-established association with tuberous sclerosis complex (TSC) and emerging evidence of rarer links to other hereditary cancer predisposition syndromes.

TSC is a hereditary cancer predisposition syndrome caused by germline mutations in the *TSC1* or *TSC2* genes. and it’s characterized by the development of benign tumors (hamartomas) in multiple organs, including the brain, skin, kidneys, heart, and lungs.^29^ Approximately 10% of PEComas arise in patients with TSC, reflecting the critical role of *TSC* gene dysfunction in tumor pathogenesis. Patients with TSC harbor germline mutations that lead to loss of heterozygosity or second-hit somatic mutations in *TSC1* or *TSC2*, resulting in constitutive activation of the mTOR pathway.^30^ This molecular mechanism underlies the development of various TSC-associated tumors, including renal AMLs, LAM, and PEComas in diverse anatomical sites. The presence of PEComa in TSC patients often correlates with multifocal disease and younger age at onset, necessitating vigilant clinical surveillance.^13^

Beyond TSC, other cancer predisposition syndromes have not been definitively linked to PEComa development; however, ongoing research aims to elucidate additional genetic factors that may contribute to susceptibility. The identification of *FLCN* mutations in some PEComa cases suggests a potential overlap with Birt-Hogg-Dubé syndrome, although this association remains rare and requires further investigation.^31^^,^^32^

Understanding the genetic basis of PEComas within the context of cancer predisposition syndromes informs risk assessment, genetic counseling, and personalized management strategies. It also highlights the importance of integrating molecular diagnostics into clinical practice to identify patients who may benefit from targeted therapies and tailored surveillance protocols.

## Diagnostic criteria and risk stratification

Given the rarity and heterogeneity of PEComas, Folpe et al.^1^ proposed to differentiating these tumors on the basis of their malignant potential. These criteria include:

1. Tumor size ≥ 5 cm
2. Infiltrative growth pattern
3. High nuclear grade and cellular atypia
4. Mitotic rate >1 per 50 high-power fields
5. Tumor necrosis
6. Vascular invasion

Tumors lacking all these features are scored as benign, while having one or more of those criteria transient the tumors from uncertain malignant to deemed malignant.

Recent studies^2^^,^^3^ have reinforced these criteria, including tumors exceeding 5 cm in diameter with necrosis or hemorrhage and evidence of capsular invasion as a higher malignancy.

In 2014, Schoolmeester et al.^33^ proposed that uterine PEComas should be classified as malignant when 4 or more malignant histologic features are present. Subsequently, Bennett et al.^16^ suggested that a lower threshold, 3 or more high-risk features, could improve the accuracy of malignancy prediction in uterine PEComas. This proposal was included into 2020 WHO Classification of Soft Tissue Tumours,^6^ which defines PEComa as follows: benign, when no high-risk features are identified; uncertain malignant potential, when 1 or 2 features are present; malignant, when 3 or more features are observed.

A recent retrospective study analyzing 87 patients with localized PEComa developed the PEC-PRO prognostic score, which integrates 4 key clinicopathologic variables: tumor size, mitotic rate, necrosis, and patient sex. This model stratifies patients into 3 risk groups with markedly different event-free survival (EFS) rates at 2 years:

- Low-risk group (score = 0): 2-year EFS of 93.7% (95% CI, 83.8-100)
- Intermediate-risk group (score = 1): 2-year EFS of 67.4% (95% CI, 53.9-80.9)
- High-risk group (score ≥ 2): 2-year EFS of 2.3% (95% CI, 0.0-18.3)

This scoring system provides a valuable tool for predicting postoperative recurrence risk and tailoring follow-up intensity.^34^

Respect to earlier models, PEC-PRO has shown superior accuracy in predicting EFS and offers a promising tool for both clinical practice and future trial stratification.

A summary of these classification systems are provided in the Tables 4 and 5.

**Table 4.** Overview of main risk stratification models for PEComas.

Risk model	Criteria	Classification outcome
**Folpe (2005)**	6 histologic features (eg, size, mitoses, necrosis)	≥2 = malignant; 1 = uncertain; 0 = benign
**Schoolmeester (2014)**	≥4 high-risk features in uterine PEComas	Binary: benign/uncertain vs malignant
**WHO 2020**	≥3 features = malignant	Benign/Uncertain Malignant Potential/Malignant
**PEC-PRO (2024)**	12-variable clinico-pathological score	Low (0)/Intermediate (1)/High (≥2) Risk

**Table 5.** Comparison of prognostic scoring systems for PEComa.

Parameter	Folpe criteria (2005)	PEC-PRO score (2022)
**Tumor size**	>5 cm	Point-based; >8 cm (HR 3.2, 95% CI, 1.5–7.9)
**Growth pattern**	Infiltrative	Included; infiltration (HR 2.5, 95% CI, 1.1–5.9)
**Nuclear grade**	High nuclear grade/high cellularity	Not scored separately; reflected in mitotic index
**Necrosis**	Presence of tumor necrosis	Included (HR 2.9, 95% CI, 1.4–7.0)
**Mitotic activity**	>1 mitosis per 50 HPF	>1/50 HPF (HR 3.1, 95% CI, 1.4–7.2)
**Vascular invasion**	Present	Not individually scored
**Risk categories**	Benign, uncertain malignant potential, malignant	Low risk (score 0), intermediate risk (score 1), high risk (≥2)

## Prognosis and treatments

The prognosis of PEComas is highly variable and depends on multiple clinicopathologic and molecular factors. Recent advances have led to the development of more refined prognostic models that better predict outcomes and guide clinical management. Contemporary survival outcomes vary markedly according to disease stage and risk category. For patients with localized PEComa treated exclusively with surgery, long-term follow-up studies report 5-year overall survival (OS) rates of approximately 75%-90%, with median OS often exceeding 10 years. In contrast, malignant PEComas with metastatic or unresectable presentations are associated with significantly worse prognoses: median OS in advanced cases is reported between 16 and 36 months, and 5-year survival is less than 30%, with up to 72% of patients developing metastases within the first year after diagnosis.^2^^,^^35^ A systematic review of 124 advanced PEComa cases reported a median OS of 60 months, with factors such as metastasis at diagnosis, lymph node involvement, and higher risk scores correlating with poorer outcomes.^27^ Unfavorable prognostic factors remain the malignant histologic variant, large tumor size (>5 cm), high mitotic activity, necrosis, and infiltrative growth pattern, all of which confer a higher risk of recurrence and metastasis.^2^ Notably, in malignant PEComa, late recurrences have been documented even after apparently successful treatment.^1^

Treatment approaches must be tailored according to PEComa subtype and clinical behavior. For benign or localized PEComas (typically small, asymptomatic renal angiomyolipomas, clear cell “sugar” tumors, or low-risk uterine lesions), active surveillance with periodic imaging is appropriate for stable, non-growing tumors without high-risk features. Surgical resection (preferably R0) is indicated for symptomatic lesions, documented growth, or proximity to critical structures.^7^

In contrast, malignant, advanced, or metastatic PEComas require aggressive multidisciplinary management. Complete surgical resection remains the cornerstone when feasible, with negative margins strongly associated with long-term disease control. For unresectable, recurrent, or metastatic disease, *mTOR inhibitors* (sirolimus, everolimus, nab-sirolimus) constitute the standard systemic therapy, leveraging the ubiquitous TSC1/2-mTOR pathway dysregulation in these tumors. Prognosis in advanced cases remains guarded despite these advances.^7^

### Surgical management

Surgery remains the mainstay of treatment for localized PEComa.^36^ Complete surgical excision with negative microscopic margins (R0 resection) is the recommended approach whenever possible, and is associated with the best long-term outcomes. Debulking procedures may be considered in the context of multifocal or life-threatening, particularly when symptomatic relief is needed or when complete resection is anatomically unfeasible.^7^

Laparoscopic and robotic approaches have been reported for the surgical treatment of PEComa, although evidence is limited to case reports due to the rarity of the disease. Minimally invasive surgery appears to be associated with reduced postoperative pain and shorter hospital stays; however, oncological outcomes remain poorly defined.^37^

In patients with TSC, PEComas tend to be multifocal and can involve multiple organs, raising unique management considerations. Surgery should be carefully weighed against the risks of morbidity, especially when lesions are asymptomatic or stable. In this context, a “watch and wait” strategy is appropriate for small, indolent tumors without high-risk pathological features, with intervention reserved for growing or symptomatic lesions.^2^^,^^6^^,^^29^

### mTOR inhibitors

mTOR inhibitors currently represent the cornerstone of therapy for patients with advanced, unresectable, or metastatic PEComas, owing to the pivotal role of TSC1 and TSC2 gene dysfunction in constitutive activation of the PI3K/AKT/mTOR signaling pathway, a key driver of tumorigenesis in these neoplasms. The mammalian target of rapamycin is a central regulator of cell growth, proliferation, metabolism, and survival, and its dysregulation promotes uncontrolled tumor growth in PEComas. Consistent with this biological rationale, mTOR inhibition has demonstrated clinically meaningful activity across both retrospective and prospective studies.

The most commonly used mTOR inhibitors in clinical practice include:

- Sirolimus (rapamycin): The first mTOR inhibitor introduced in oncology and immunosuppression, sirolimus has shown clinically meaningful activity in PEComas, with tumor shrinkage and prolonged disease stabilization reported in early case series, particularly in TSC-associated tumors. A 10-year real-world experience by Świtaj et al. further confirmed sustained responses and disease control in a significant proportion of patients.^38^
- Everolimus: A sirolimus derivative with improved pharmacokinetic properties, everolimus has demonstrated significant antitumor activity in clinical studies and case series, with durable responses and manageable toxicity. It is approved for several TSC-related tumors^39^ and is widely used in PEComas harboring TSC1/TSC2 alterations.^7^
- Nab-sirolimus (ABI-009) is an albumin-bound nanoparticle formulation designed to enhance tumor delivery and bioavailability; in the phase II AMPECT trial,^40^ conducted in patients with advanced malignant PEComas, it demonstrated particularly promising results, with an objective response rate of 39% and a median progression-free survival of 10.6 months, leading to its regulatory approval. TSC2 mutations were enriched among responders (85% of the cohort), with higher response rates observed in these patients, supporting a biomarker-driven therapeutic approach; however, responses were also seen in patients with TSC1 alterations or no TSC1/TSC2 mutations, highlighting the value of molecular profiling and supporting the use of nab-sirolimus across diverse molecular subgroups.^22^^,^^36^^,^^41^

In addition to mTOR inhibition, antiangiogenic agents targeting VEGFR, such as pazopanib and sunitinib, have shown modest activity in small case series and may represent alternative options in selected patients, although the level of evidence remains limited.^35^^,^^38^^,^^41–43^

The mTOR inhibitors bind to the intracellular receptor FKBP12, forming a complex that allosterically inhibits mTORC1, thereby suppressing downstream signaling pathways involved in protein synthesis, angiogenesis, and cell cycle progression. This inhibition leads to decreased tumor cell proliferation, induction of autophagy, and apoptosis. However, mTORC2 is generally not inhibited by these agents, which may contribute to resistance mechanisms.^44^

Multiple retrospective studies, case reports, and prospective trials have established the efficacy of mTOR inhibitors in PEComa. Patients treated with sirolimus or everolimus frequently experience partial responses or disease stabilization, with improvements in symptoms such as pain and organ dysfunction. Treatment is generally well tolerated; common adverse effects include mucositis, hyperlipidemia, rash, and mild immunosuppression.^25^^,^^38^^,^^45^

A summary of the main cases treated reported in the literature are presented in Table 6.

**Table 6.** Summary of reported studies and case series.

Study	Evidence type	Number	Setting/site	Main outcome
**SIROLIMUS**				
** Wagner et al., 2010^41^**	Case series	3	Metastatic PEComas	Tumor regression in all patients
** Dickson et al., 2013^46^**	Case series	5 (11 pooled)	Extrarenal PEComas	3 CR, 1 PR, 1 PD (ORR 80%)
** Scheppach et al., 2013^47^**	Case report	1	Colon PEComa	PD after 4 months
** Bergamo et al., 2014^48^**	Case report	1	Hepatic PEComa	Tumor shrinkage → surgery
** Ghosh et al., 2014^49^**	Case report	1	Pelvic PEComa	PR
** Benson et al., 2014^35^**	Retrospective series	10	Advanced PEComa	5 PR, 1 SD, 1 PD
** Sun et al., 2015^50^**	Case report	1	Uterine PEComa	PR
** Batereau et al., 2016^51^**	Case reports	2	Peritoneal/iliac	Both CR
** Gao et al., 2016^52^**	Case report	1	Advanced PEComa	Rapid response
** Liu et al., 2016^53^**	Case report	1	Small intestine	PD
** Starbuck et al., 2016^54^**	Case series	3	Uterine PEComa	2 responses, 1 PD
** Tang et al., 2017^55^**	Case report	1	Abdominal wall	PD
** Varan et al., 2017^56^**	Case report	1	Orbital	PD
** Westaby et al., 2017^57^**	Case report	1	Ovarian	PD
** Raimondi et al., 2018^58^**	Case report	1	Renal PEComa	PR (18 months)
** Gondran et al., 2019^59^**	Case report	1	Pancreatic PEComa	PR (∼42 months)
** Sanfilippo et al., 2019^43^**	Case series	7	Advanced PEComa	DCR 86%
** Rao et al., 2020^60^**	Case report	2	Pelvic/cardiac	CR + SD
** Świtaj et al., 2021^38^**	Retrospective series	15	Systemic PEComa	ORR 73%
** Leach et al., 2025^61^**	Case report	1	Metastatic PEComa	PD
**NAB-SIROLIMUS**				
** Wagner et al., 2021 (AMPECT)^25^**	Phase II trial	31	Advanced PEComa	ORR 39%, durable responses
** Meredith et al., 2023^62^**	Case report	1	Mesenteric PEComa	Clinical benefit
** Palma et al., 2023^63^**	Subgroup analysis	16	Gynecologic PEComa	Confirmed activity
** Wagner et al., 2024^40^**	Phase II update	31	Same cohort	DOR 39.7 mo; OS 53.1 mo
** Targeted Oncology, 2024^64^**	Subgroup report	16	Female PEComa	ORR 37.5%
**EVEROLIMUS**				
** Gennatas et al., 2012^42^**	Case report	1	Retroperitoneal	Clinical response
** Dickson et al., 2013^46^**	Case series	1	Hepatic	PR → surgery
** Chen et al., 2014^65^**	Case report	1	Pelvic	SD
** Weeber et al., 2015^66^**	Case report	1	Metastatic	Clinical benefit
** Binyamin et al., 2016^67^**	Case report	1	Renal	PD
** Flechter et al., 2016^68^**	Case report	1	Small bowel	SD
** Shaikh et al., 2016^69^**	Case report	1	Extraperitoneal	PR (∼7 months)
** Kwon et al., 2017^70^**	Case report	1	Uterine/vaginal	SD (18 months)
** Hulova et al., 2018^71^**	Case report	1	Renal	SD (∼30 months)
** Saluja et al., 2018^72^**	Case report	1	Head/neck	PD
** Purwar et al., 2022^73^**	Case report	1	TFE3 pelvic	CR
** Sui et al., 2022^74^**	Case report	1	Uterine metastatic	PR, PFS 15 mo
** Zhang et al., 2022^75^**	Case report	1	Renal	Tumor remission
** Yang et al., 2024^76^**	Case report	1	Hepatic/renal (TSC)	Good postoperative control
** Zhong et al., 2025^77^**	Case report	1	Uterine metastatic	Durable remission (∼3 years)
** Saliba et al., 2025^78^**	Case report	1	Pulmonary embolic PEComa	PR
** Kitagawa et al., 2025^79^**	Case report	1	Hepatic aggressive	PD
**VEGFR inhibitor**				
** Wagner et al., 2010 (sunitinib)^41^**	Case series	1 (subset)	Metastatic PEComa	Limited activity; inferior to sirolimus
** Benson et al., 2014 (sunitinib)^35^**	Retrospective series	subset	Advanced/metastatic PEComa	Minimal activity; no objective responses
** Gennatas et al., 2012 (sunitinib)^42^**	Case report	1	Metastatic PEComa	No significant response
** Sanfilippo et al., 2019 (pazopanib)^43^**	Case report	1	Metastatic uterine PEComa	Disease stabilization
** Zhang et al., 2022 (apatinib)^75^**	Case report	1	Progressive renal PEComa	PD; later response to everolimus

### Checkpoint inhibitors

Emerging evidence supports the role of immune checkpoint inhibitors (ICIs) in malignant PEComas, particularly those exhibiting high tumor mutational burden (TMB) and elevated programmed death-ligand 1 (PD-L1) expression.^80–83^ Case reports have documented durable responses to pembrolizumab, alone or combined with everolimus, in metastatic PEComa patients harboring high TMB (eg, >10 mutations/Mb) and PD-L1 tumor positivity scores exceeding 1%, with some achieving near-complete remission.^84–86^ These findings suggest that immunotherapy may represent a valuable option for selected patients, especially those refractory to mTOR inhibitors or chemotherapy.

However, immune-related adverse events, including severe toxicities such as myocarditis, have been reported with immune checkpoint inhibitors, underscoring the need for careful patient selection and close monitoring. Moreover, the immunologically “cold” tumor microenvironment characteristic of PEComas may limit response rates; nevertheless, combination strategies with mTOR inhibitors or radiotherapy could potentially enhance therapeutic efficacy.

### Locoregional therapies

In selected cases, locoregional treatment modalities may be considered for unresectable or symptomatic PEComas.

Embolization can be used to manage hepatic or large pelvic tumors, either to control bleeding or reduce tumor bulk prior to surgery.^87^ Embolization and everolimus are established specifically for large renal angiomyolipomas (>3-4 cm) at risk of spontaneous hemorrhage, primarily in TSC patients. There is no routine indication for embolization or systemic therapy in smaller or non-renal benign PEComas unless clinically symptomatic (eg, significant mass effect or organ dysfunction).^88^

Radiotherapy plays a supportive role in PEComa management, primarily for palliation of symptoms or local control in unresectable or recurrent tumors. Although PEComas are generally considered radioresistant, radiotherapy has demonstrated efficacy for tumor shrinkage and symptom management in select cases.^7^^,^^89^ Notably, in a reported case of metastatic perirectal PEComa, radiotherapy was combined with pembrolizumab and everolimus, resulting in durable disease control and symptomatic improvement.^85^ Additionally, selective internal radiation therapy (SBRT) or radioembolization has been applied to liver metastases with encouraging results.^90^

### Chemotherapy

Conventional chemotherapy has limited efficacy in PEComas and is generally reserved for refractory or rapidly progressive disease. Doxorubicin-based regimens have been used with variable success, but no standardized chemotherapy protocols exist due to the rarity of the disease.^2^^,^^7^^,^^91^

## Future perspectives and the need for collaborative international efforts

Currently, international evidence derives mainly from retrospective series or single-arm prospective studies, limiting the robustness and generalizability of available data. There is a critical need for large, multicenter, prospective cohort studies, and the establishment of international biobanks to facilitate molecular, prognostic, and therapeutic research in both sporadic and syndrome-associated PEComas.

Despite the ultrarare nature of PEComa and its variable clinical behavior, international collaboration through biobanking and data sharing is essential. Priority should be drawn to define predictive biomarkers of therapy responses, as well as comprehensive genomic and transcriptomic profiling from patient-derived tumors, are needed to deeply investigate tumor biology and molecular therapy driven.

International efforts should primarily focus on strongly encouraging the reporting of patients to the NORD registry (National Organization for Rare Disorders),^92^ to the EORTC (European Organisation for Research and Treatment of Cancer),^93^ formerly to the PEComa & Rare Sarcoma Working Group, and to the national rare tumor registries. Such collective efforts are crucial for generating higher-quality evidence to realize clinical trials or develop consensus guidelines needed to improve patient care.

## Discussion

PEComas represent a rare and heterogeneous group of mesenchymal neoplasms with unique histopathological and molecular characteristics. This review consolidates current knowledge on their epidemiology, molecular biology, diagnostic criteria, treatment options, and prognosis, highlighting both advances and ongoing challenges in managing this ultra-rare tumor family.

The rarity of PEComas and the relatively recent identification (first description nearly 30 years ago), cause significant challenge to large-scale epidemiological studies and clinical trials. Most available data derive from case reports, small retrospective series, and limited prospective studies, underscoring the need for multicenter collaborations and registries to better define disease behavior and optimize management strategies.

Molecular insights have revolutionized our understanding of PEComa pathogenesis and the involvement of the mTOR pathway via *TSC1*/*TSC2* gene^2–4^^,^^14^ permitted the introduction of with the mTOR inhibitors as the mainstay of treatment for advanced or unresectable disease. The recent clinical success of nab-sirolimus,^25^^,^^40^ with high response rates in *TSC2*-mutated tumors, marks a significant therapeutic breakthrough. However, the existence of *TFE3*-rearranged PEComas,^10^^,^^11^^,^^19^^,^^26^ which likely follow an mTOR-independent oncogenic pathway, highlights the molecular heterogeneity and the necessity for tailored treatment approaches.

Histopathological evaluation remains fundamental for diagnosis and risk stratification. Folpe’s criteria^1^ and the newer PEC-PRO prognostic score^34^ offer valuable tools for predicting malignant potential and recurrence risk, facilitating individualized patient management. Surgical resection remains the cornerstone for localized tumors,^36^ but systemic therapies are essential for advanced cases in which prognosis remains dismal.^7^ Immune checkpoint inhibitors might be a possible alternative in case with loss of mTOR-I responses or in selected patients with high tumor mutational burden and PD-L1 expression, although immune-related toxicities must be carefully considered.^80^^,^^81^ Nonetheless, optimal treatment strategies are still evolving, and novel therapeutic approaches such as tyrosine kinase inhibitors and immune checkpoint blockade are under investigation.

Survival in PEComa is highly variable, with 5-year overall survival (OS) rates of 75%-90% and median OS exceeding 10 years in patients with localized, resected disease, contrasted with a median OS of only 16-36 months and less than 30% 5-year survival in advanced malignant cases.^2^^,^^27^^,^^35^ These data underscore the profound prognostic influence of disease stage and histologic aggressiveness.

The scope of this review is to stimulate the international community involved in soft tissue neoplasm and in general in ultra-rare disease to run future research and prospective clinical trials, including molecular characterization, identification of new predictive biomarkers, the discovery of novel therapeutic drugs targeting alternative pathways in the cases of mTOR-independent PEComa.

## Conclusion

Perivascular epithelioid cell tumors are ultra-rare mesenchymal neoplasms with distinctive histopathological features and significant molecular heterogeneity. The identification of TSC1/TSC2 inactivation and consequent mTOR pathway activation has improved disease understanding and established mTOR inhibitors, including nab-sirolimus, as the mainstay of treatment for advanced disease. However, TFE3-rearranged PEComas represent a distinct subset requiring mTOR-independent therapeutic approaches. Histopathology remains central to diagnosis and risk stratification, with Folpe’s criteria and the PEC-PRO score aiding prognostic assessment. Surgery is the treatment of choice for localized disease, while outcomes in advanced settings remain poor. International collaboration, prospective registries, and integrated molecular profiling are essential to advance personalized treatment strategies.
